# Effect of light on the growth and photosynthesis of an invasive shrub in its native range

**DOI:** 10.1093/aobpla/plu033

**Published:** 2014-06-26

**Authors:** Maya Svriz, María A. Damascos, Karen D. Lediuk, Santiago A. Varela, Daniel Barthélémy

**Affiliations:** 1 Departamento de Botánica, Centro Regional Universitario Bariloche, Universidad Nacional del Comahue, Quintral 1250, 8400 Bariloche, Argentina; 2 Concejo Nacional de Investigaciones Científicas y Técnicas (CONICET), C1033AAJ, Ciudad Autónoma de Buenos Aires, Argentina; 3 INTA Estación Experimental Agropecuaria Bariloche, CC 277, 8400 Bariloche, Argentina; 4 CIRAD, BIOS, Direction and INRA, UMR AMAP, F-34398 Montpellier, France

**Keywords:** *Berberis darwinii*, ecophysiological attributes, light environments, native and invasion area, plant invasion.

## Abstract

Invasive species' success may depend on ecophysiological attributes present in their native area or those derived from changes that took place in the invaded environment. We studied the growth and photosynthetic capacity of *Berberis darwinii* shrubs growing under different light conditions (gap, forest edge and below the canopy) in their native area of Patagonia, Argentina. Leaf photosynthesis results determined in the native area were discussed in relation to information provided by studies carried out under the same light conditions in an invaded area in New Zealand. Shoot elongation, leaf production, stem and leaf biomass per shoot, and specific leaf area (SLA, cm^2^ g^−1^) were determined in five adult plants, randomly selected in each of the three light conditions at two forest sites. Net photosynthesis as a function of PPFD (photosynthetic photon flux density), stomatal conductance (*g*_s_), maximum light-saturated photosynthesis rate (*P*_max_), *P*_mass_ (on mass bases) and water-use efficiency (WUE_i_) were determined in plants of one site. We predicted that functional traits would differ between populations of native and invasive ranges. In their native area, plants growing under the canopy produced the longest shoots and had the lowest values for shoot emergence and foliar biomass per shoot, while their SLA was higher than gap and forest edge plants. Leaf number and stem biomass per shoot were independent of light differences. Leaves of gap plants showed higher *P*_max_, *P*_mass_ and *g*_s_ but lower WUE_i_ than plants growing at the forest edge. In its native range *B. darwinii* grows under different light conditions by adjusting shoot and leaf morphology and physiology. Plants of *B. darwinii* growing under the same light conditions show similar physiology in native and invasive ranges. This means that for *B. darwinii*, intra-specific variation of the functional traits studied here does not condition successful spread in new areas.

## Abstract (precis)

We studied the growth and photosynthetic capacity of *Berberis darwinii* shrub growing under different light conditions in their native area of Argentina to test if plant physiology differs from invaded area (using studies carried out in New Zealand). In its native range *B. darwinii* grows under different light conditions by adjusting shoot and leaf morphology and physiology. Plants of *B. darwinii* growing under the same light environments show similar physiology in native and invasive ranges. Therefore, intra-specific variations of the functional traits in native area do not condition successful invasiveness.

## Introduction

Studies of the successfulness of invading exotic species commonly focus on plant attributes and environmental factors that control their persistence in the invaded area, but less information is available on populations of the same species growing in its native area (Erfmeier and Bruelheide 2004, 2005; Hierro *et al.* 2005). One main objective is to establish whether the properties that determine the survival and spread of an exotic species in their invasion range are inherent to the species (i.e. are already present in native area populations) or represent changes in plant traits in the new, invaded ranges (Güsewell *et al.* 2006). Comparative studies have determined that populations of species growing in invaded areas exhibit higher plant density and dominance (Moroney and Rundel 2012), size (Crawley 1987; Willis *et al.* 2000) and growth rates, as well as the presence of shorter-lived leaves (Erfmeier and Bruelheide 2004) than in their native areas. Exotic invasive *Rubus* species showed higher photosynthetic capacity, maximum photosynthesis, water and nitrogen use efficiency than native *Rubus* growing in the same areas (McDowell 2002).

Light availability, which varies both spatially and temporally (Horn 1971) and exhibits qualitative and quantitative heterogeneity (Valladares 2003), limits the establishment of forest species, determining differences in plant growth and in physiological and morphological plant responses (Sims and Pearcy 1992; Poorter 1999; Valladares and Niinemets 2008).

*Berberis darwinii* Hook. (Berberidaceae) is a spiny evergreen shrub native to southern Argentina and the Chilean Andean forests, and is an introduced invasive species in New Zealand (Allen 1991), Great Britain, Australia and the USA (USDA 2006). In its native area of Chiloé (Chile), a wetter region than its distribution range in Argentina, high seed germination and seedling growth occurs both under the forest canopy and in gaps with canopy openness greater than 20 % (Figueroa and Armesto 2001; Figueroa and Lusk 2001; Figueroa 2003). In New Zealand *B. darwinii* can become established and persist below the forest canopy (Allen 1991; Allen and Wilson 1992). According to McAlpine and Jesson (2007), seedlings of this species are shade intolerant, while adult plants growing under the canopy come from surviving seedlings with increased shade tolerance. However, although this shrub can grow in both low and high light levels, it achieves higher performance (seedling establishment, total biomass, maximum photosynthesis) in the latter conditions, outperforming even coexisting native species (McAlpine 2005; McAlpine *et al.* 2008).

The photosynthetic responses of *B. darwinii* leaves to different light levels and other ecophysiological characteristics have been studied extensively in invaded areas of New Zealand (Allen 1991; Allen and Wilson 1992; McAlpine 2005; McAlpine and Jesson 2007; McAlpine *et al.* 2008).

In the present work we studied growth and biomass allocation to stems and leaves in current-year shoots and variation in photosynthesis, instantaneous water-use efficiency (WUE_i_) and leaf morphology in adult plants of *B. darwinii* growing under different light conditions in the evergreen temperate forests of the Andean region of Patagonia, Argentina. We hypothesized that adult plants of *B. darwinii* populations in Argentina would exhibit differences in some characteristics in response to different forest light levels. We predicted that plants growing in gaps would have, for example, greater shoot growth, biomass and leaf photosynthetic activity than plants growing at the forest edge and in the understorey. Photosynthetic parameters (estimated from photosynthesis models; see below) and specific leaf area (SLA, cm^2^ g^−1^) values obtained in the native area studied were compared with those of the invaded area (bibliographic data) for the same light environments. We predicted that although the *B. darwinii* populations in the native and invaded areas (New Zealand) might show a similar pattern of variation in relation to different light environments in the forest, the physiology of the introduced populations would differ from that of plants growing in their native range.

## Methods

### Study area

The study was conducted at two sites located on Victoria Island in Nahuel Huapi lake, within the Nahuel Huapi National Park in Neuquen province, Argentina. Mean annual precipitation in the area is 1700 mm (Barros *et al.* 1983). The soils are derived from volcanic ash; they are sandy, acidic and rich in organic matter (Koutche 1942).

At both study sites (Site 1: 40°57.94′S, 70°31.34′W, 791 m.a.s.l.; Site 2: 40°59,02′S, 71°31.33′W, 790 m.a.s.l.) the forest is dominated by the native evergreen species *Nothofagus dombeyi* (Mirb.) Oerst (Fagaceae).

**Figure 1.**
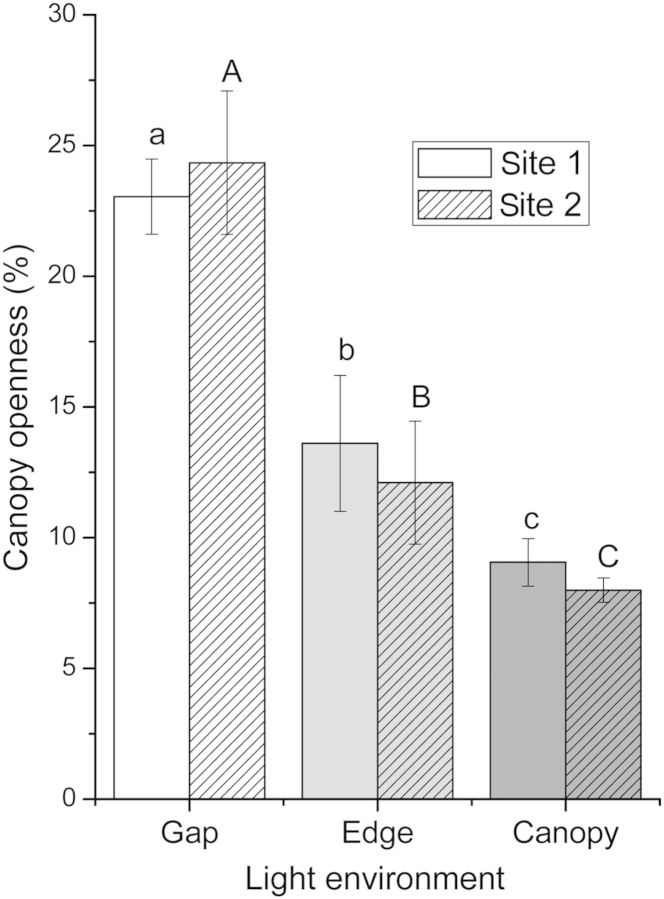
Canopy openness (mean ± SE) in the gap, at the forest edge and under the canopy of the *N. dombeyi* forest at the two sampling sites. Different lowercase letters indicate significant differences between the three light environments compared at Site 1; for the same comparison at Site 2, different uppercase letters are used. Two-way ANOVA.

**Figure 2.**
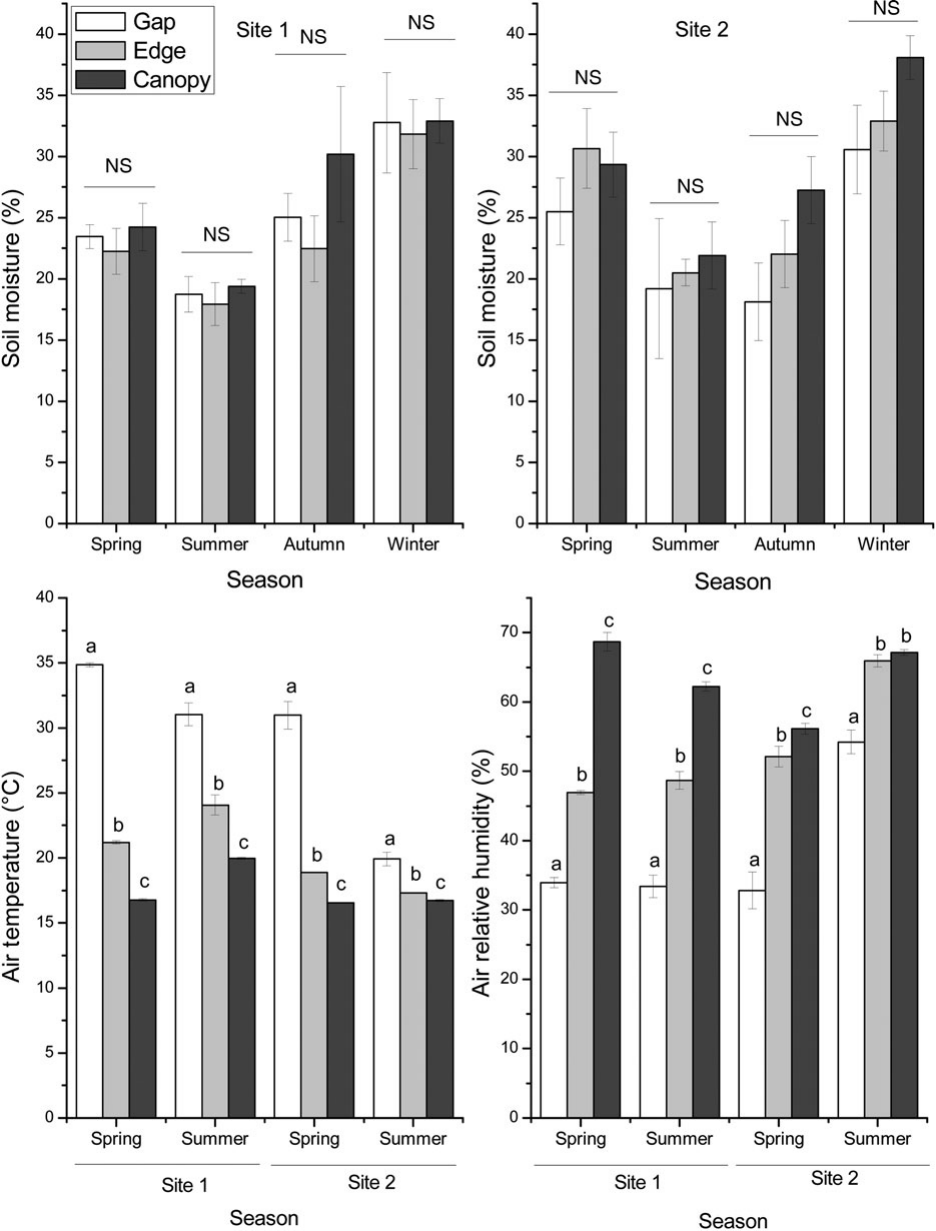
Mean percentage (±SE) of soil moisture, air temperature and relative humidity in the gap, at the edge and under the canopy of the *N. dombeyi* forest at two sites on Victoria Island, Argentina, corresponding to the native area. NS indicates no significant differences. Different lowercase letters indicate significant differences between light environments in each season.

Study sites were selected based on the existence of a forest gap caused by clearing, with *B. darwinii* present both in the gap and in surrounding areas. The gap size was 1496 m^2^ at Site 1 and 2020 m^2^ at Site 2. Light availability was estimated from the percentage of canopy openness (Fig. 1), determined by hemispherical photographs and analysed with the GLA program (Gap Light Analyzer, version 2.0: imaging software). Light availability differed between forest gap, edge and under the canopy area (two-way ANOVA, *P* ≤ 0.001), while comparisons between sites (*P* = 0.563) and the interaction (*P* = 0.249) were not significant. Both air temperature and relative air humidity (%RH) measured during spring and summer differed between light environments at each site (*P* ≤ 0.001; Fig. 2).

Seasonal soil moisture values did not differ between the three light environments at each site (Fig. 2), possibly due to the existence of a 10-cm layer of volcanic ash on the ground during sampling, a result of the Puyehue volcano eruption in June 2011, which prevented soil water evaporation.

Soil nutrient availability did not differ between disturbed forest areas located near plantations and the native undisturbed forests on Victoria Island (Nuñez *et al.* 2009).

### Species description

*Berberis darwinii* is a shrub that grows up to 2 m in height and produces yellow-orange flowers in racemose inflorescences and blue-black berries (Brion *et al.* 1998). Fasciculate leaves are four in number. In Argentina this species is widely distributed in forests of the Andean region of Neuquen, Rio Negro and Chubut provinces (Orsi 1984). It grows in forests of *Nothofagus pumilio* (Poepp. and Endl.) Krasser (Fagaceae), *N. dombeyi* and *Austrocedrus chilensis* (D. Don) Pic. Serm. and Bizzarri (Cupressaceae), in shrublands of *Nothofagus antarctica* (G. Forst.) Oerts (Damascos 2005) and in the Valdivian rainforest of Puerto Blest (Brion *et al.* 1998). Albeit with low coverage, it grows in disturbed areas in shrublands of the exotic species *Rosa rubiginosa*, Rosaceae (Svriz 2008). In Chile it grows from 33°25′ to 46°40′S, between 150 and 1300 m.a.s.l., at similar latitudes to Argentina (Landrum 1999). On Chiloe Island, Chile, *B. darwinii* grows at the edges of the Valdivian forest and at open sites together with secondary vegetation (Figueroa and Armesto 2001). According to germination requirements, *B. darwinii* would appear to be a generalist species with regard to light, but when seedling survival is considered this species seems to prefer intermediate–high light conditions (Figueroa and Lusk 2001).

### Sampling

Gap, edge and area under the canopy were delimited at each site using a global positioning system. Edge area corresponded to the projection of the tree canopy surrounding the gap, from the outer limit of the latter and to a distance of 5m into the forest. Canopy area was defined as being from the internal edge limit into the forest.

Current-year shoot production, leaf and stem shoot biomass and growth as well as leaf traits were measured on plants from the two sampling sites, while photosynthesis (see below) was evaluated only at Site 1.

During spring (mid-October 2011), at each site and for each light environment (gap, edge and under the forest canopy), five *B. darwinii* plants between 0.5 and 1.50 m height were randomly selected. Since apex abscission occurs in long shoots at the end of the growing season (M.S., unpubl. res.), three lateral buds located immediately below the apex were labelled on two previous-year shoots per plant. Every 15 days the number of buds producing shoots was quantified, stem elongation was measured using a caliper, and the number of leaves on each shoot emerging from the labelled buds was counted.

In February 2012 (summer) following the cessation of current-year shoot extension, the net leaf photosynthesis (*P*_n_; µmol CO_2_ m^−2^ s^−1^) curves as a function of PPFD (photosynthetic photon flux density) were obtained for the same five individuals considered for growth measurements at Site 1. Measurements were performed during mid-morning using a gas exchange infrared Li-cor 6400 (Lincoln, NE, USA) with PPFD values of 10, 50, 100, 200, 500, 1000 and 1500 µmol photons m^−2^ s^−1^. Each measurement was made using a minimum waiting time of 180 s at each light intensity, under controlled conditions of air temperature (19 °C) and relative humidity (35 %). Leaf-to-air vapour pressure deficit was held between 1.0 and 1.5 kPa, and sample CO_2_ concentration was 400 µmol mol^−1^. Photosynthesis was measured on healthy, expanded leaves present on the upper third of a shoot selected on each individual growing in the gap, at the edge and below the forest canopy.

For SLA (cm^2^ g^−1^) determination, we used samples of 50 healthy leaves taken from each of the five *B. darwinii* individuals per site and light environment. One leaf disc (of known area) per blade was cut away from the midrib (Cornelissen *et al.* 2003). Leaf circles were dried in an oven at 70 °C for 3 days until constant dry weight. Leaf mass per area (LMA, g m^−2^) was calculated from the same data.

During the same period, current-year shoots (the shoots that emerged from labelled buds, and of which stem elongation had been measured and leaf production counted) were cut. The stem and leaves of each one were separated and dried using the method mentioned above for determination of dry biomass.

### Statistical analyses

Since sites did not differ significantly in the canopy openness of each light environment (gap, edge and area below the forest canopy) the data obtained from the plant variables studied at each site were pooled, so that 10 measurements of each variable per light environment were obtained.

The proportion of labelled axillary buds producing shoots in spring was compared between plants growing in the three light environments using a chi-square test. Shoot elongation during the growing period was fitted to Holling type-III function (Bolker 2007) for plants of each light environment, as follows:

$$y=ax^{2}/b^{2}+x^{2}$$

where *y* is the accumulated shoot growth (cm), *x* is the time, *a* is the maximum accumulated growth value (cm) and *b* represents the time at the half-maximum accumulated growth (Bolker 2007). Data fitting was performed using nonlinear regression in Prism4 (GraphPad, San Diego, CA, USA). The accuracy of the fitted parameters was examined via the ratios between the standard errors of estimate (SEE) and the best fitted values (Zar 1999). We used global fitting (Motulsky and Christopoulus 2004) to compare the fitted parameters between different light conditions. In each case we report the statistic evidence ratio (ER) in favour of the better model (i.e. global vs. separate fitting to the data; Motulsky and Christopoulus 2004).

Leaf and node numbers, internode length, and stem and leaf biomass per shoot were compared between gap, edge and below canopy growing plants using the Kruskal–Wallis test. Mean values of SLA were compared between plants of the three forest environments studied using the same procedure, while LMA was compared with a one-way ANOVA.

In order to correlate net leaf photosynthesis (*P*_n_, µmol CO_2_ m^−2^ s^−1^) as a function of photosynthetic flux density (PPFD) the dataset was fitted to a non-rectangular hyperbola (Marshall and Biscoe 1980; Cannell and Thornley 1998) as follows:

$$P_{n}=\{\alpha I_{i}\text{1/2}\theta +P_{\max}-[(\alpha I_{i}+P_{\max}{)}^{2}-4\theta \alpha I_{i}P_{\max}{]}^{1/2}\}$$

where *P*_max_ is the maximum light-saturated photosynthesis rate, *I*_i_ is the incident radiation (µmol m^−2^ s^−1^ PPFD), *α* is the apparent quantum yield or radiation use efficiency (µmol CO_2_ mol^−1^ PPFD) and *θ* is the angle of curvature (dimensionless). Model and parameter fitting were performed using the same methods used for growth model fitting. We estimated photosynthetic rate on a per unit mass basis from the light-saturated photosynthetic rate and SLA, and this was compared between plants growing in the gap, at the edge and below the forest canopy conditions using a one-way ANOVA. Instantaneous water-use efficiency (µmol CO_2_ mol H_2_O^−1^) was estimated as the ratio between *P*_n_ at saturating PPFD and transpiration (*E*, mol H_2_O m^−2^ s^−1^), and compared between leaves of plants growing in different light environments using the Kruskal–Wallis test. Stomatal conductance (*g*_s_, mol H_2_O m^−2^ s^−1^) was compared between plants growing in the gap, at the edge and below the forest canopy using a one-way ANOVA.

## Results

### *Berberis darwinii* shoot production and growth in different light environments

**Figure 3.**
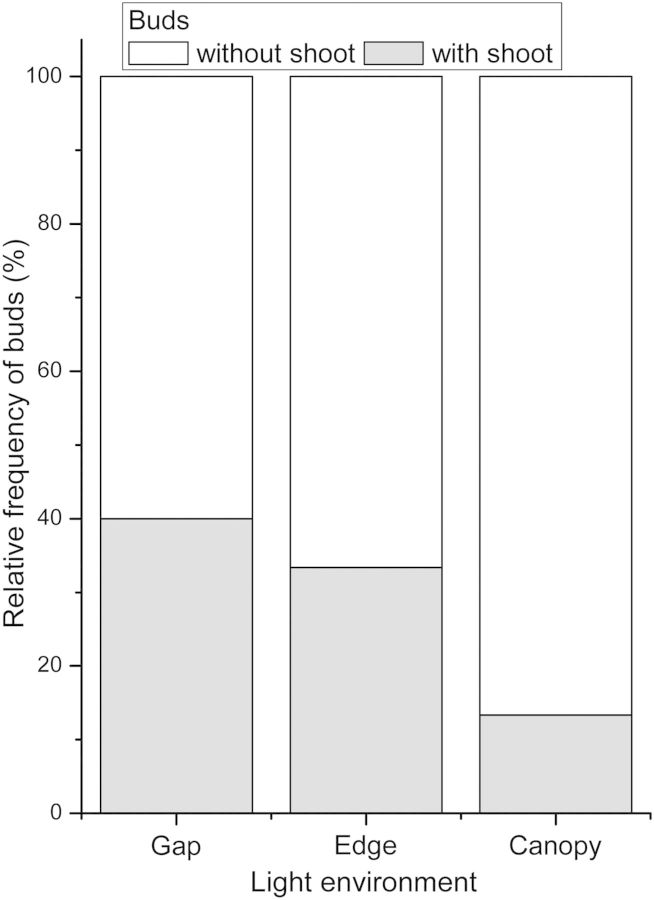
Relative frequency of marked buds with or without shoot production in *B. darwinii* plants present in the gap, at the edge and under the canopy of the *N. dombeyi* forest. Chi square test.

Labelled buds in previous-year shoots of plants growing under the canopy produced the lowest number of new shoots, values for gap and forest edge plants being twice as high (χ^2^=11.25, *P* = 0.004; Fig. 3).

**Figure 4.**
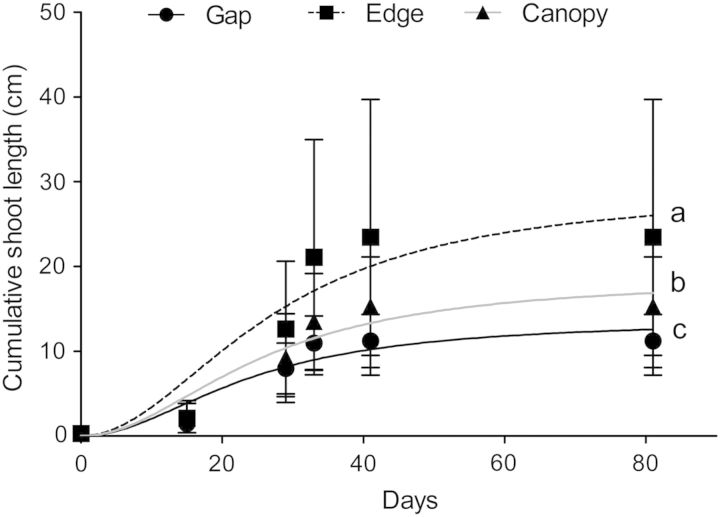
Mean (±SE) values for shoot growth (cumulative length, in cm) in the *B. darwinii* plants present in the gap, at the edge and under the canopy of the *N. dombeyi* forest as a function of time. Distinct letters indicate significant differences between light environments analysed using the global-fitting technique.

The shoot extension period in plants in all three light environments lasted ∼40 days (Fig. 4). The Holing type III function fit the cumulative shoot growth data well, with *R*^2^ values of 0.70, 0.40 and 0.60 for plants growing in the gap, at the edge and under the canopy, respectively (Fig. 4). Shoot growth differed between the three light environments (Fig. 4), where ER tends to infinity. Statistically significant differences were found between total shoot elongation values (*a*) of gap (13.67 ± 0.88 cm), forest edge (29.02 ± 4.18 cm) and under canopy (18.59 ± 2.12 cm) plants, with the following values: ER_(edge vs. gap)_ = 645; ER_(edge vs. under canopy)_ = 2.12 and ER_(gap vs. under canopy)_ = 22.74. However, time at the half-maximum accumulated growth (*b*) (gap = 23.81 ± 2.61 days; forest edge = 27.50 ± 6.11 days; under the canopy = 25.76 ± 4.76 days; ER_(edge vs. gap)_ = 2.52; ER_(edge vs. under canopy)_ = 2.87; ER_(gap vs. under canopy)_ = 2.60) did not differ significantly.

Mean shoot internode length was lower in plants from gaps than in those of the other two light environments, while the leaf and node number did not differ between plants present in the gap, at the forest edge or under the canopy (Table 1).

**Table 1.** Mean values (±SE) for leaf number, node, internodes length and stem biomass, leaves of current year leaf mass per area (LMA), in plants of *B. darwinii* growing in the gap, at the edge and under the canopy of the *N. dombeyi* forest. Distinct letters indicate statistically significant differences between light environments. Kruskal–Wallis one way analysis of variance on ranks. *P*, associated probability.

Shoot	Light environment			*P*
	Gap	Edge	Canopy	
Leaves number	31.70 ± 2.69^a^	37.53 ± 5.47^a^	25.44 ± 2.74^a^	0.351
Node number	9.11 ± 0.49^a^	12.26 ± 2.22^a^	8.44 ± 0.88ª	0.507
Internode length (cm)	1.23 ± 0.05^a^	1.88 ± 0.11^b^	1.80 ± 0.10^b^	<0.001
Stem biomass (g)	0.14 ± 0.018^a^	0.43 ± 0.17^a^	0.12 ± 0.05^a^	0.260
Leaves biomass (g)	0.69 ± 0.08^a^	0.77 ± 0.15^a^	0.33 ± 0.09^b^	0.016
LMA (g m^−2^)	196.78 ± 6.57	131.57 ± 6.53	87.99 ± 7.50	<0.001

### Leaf photosynthesis—light response

**Figure 5.**
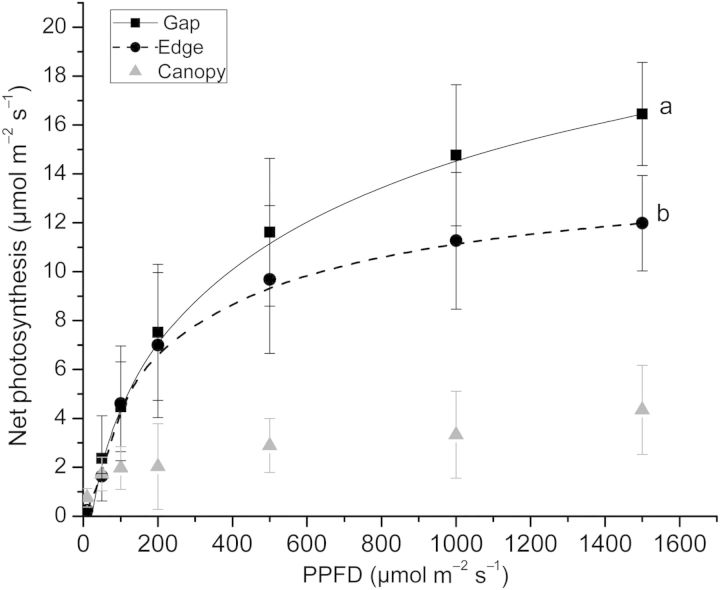
Adjusted curves of net photosynthesis as a function of PFFD (photosynthetically active radiation) of leaves in *B. darwinii* plants growing in the gap and at the *N. dombeyi* forest edge. Distinct letters indicate significant differences between models of the light environments considered, analysed using the global-fitting procedure.

Only the *P*_n_–PPFD curves of gap and forest edge plants fitted the Cannel and Thornley model, showing *R*^2^ values of 0.89 and 0.81, respectively, and they were significantly different (ER = 37.67; Fig. 5). The *α* and *θ* parameters did not differ between the two light environments, while *P*_max_ was higher in leaves of gap plants than in those of forest edges (Table 2). The photosynthetic rate on a per-unit mass basis was higher in leaves of gap and edge forest plants than in those of under forest canopy (*P* = 0.026; Table 2).

**Table 2.** Maximum light-saturated photosynthesis rate (*P*_max_), quantum yield (*α*), curvature angle (*θ*), water-use efficiency (WUE_i_), stomatal conductance (*g*_s_) and photosynthetic rate on a per-unit mass basis (*P*_mass_) for leaves of *B. darwinii* plants present in the gap, at the edge and under the forest canopy of the *N. dombeyi* forest. Distinct letters indicate significant differences and similar letters indicate no significant differences between gaps, forest edge and under canopy plants. The first three variables (*P*_max_, *α* and *θ*) were compared with the global adjustment technique and the evidence ratio (ER) in favour of the better model (i.e. global vs. separate fitting to the data) while WUE_i_, *g*_s_ and *P*_mass_ were analysed using a one-way ANOVA.

Photosynthetic parameters	Light environment			ER
	Gap	Edge	Canopy	
*P*_max_ (µmol CO_2_ m^−2^ s^−1^)	17.09 ± 1.44a	12.09 ± 1.1b	5.13 ± 0.01	13.62
*α* (µmol CO_2_ µl mol PPFD^−1^)	0.054 ± 0.02a	0.054 ± 0.02a	1.07×10^−17^±3.77×10^−16^	3.76
*θ* (dimensionless)	0.47 ± 0.89a	0.1 ± 1.23a	0.05 ± 0.006	3.61
WUE_i_	65.57 ± 3.50a	138 ± 32.56b	82.36 ± 3.61ab	
*g*_s_ (mol H_2_O m^−2^ s^−1^)	0.22 ± 0.03a	0.09 ± 0.03b	0.05 ± 0.006b	
*P*_mass_ (µmol CO_2_ g^−1^ s^−1^)	8×10^−2^ ± 6 × 10^−3^a	8.5×10^−2^ ± 1.5×10^−2^a	0.04 ± 0.08b

The WUE_i_ was significantly higher in the leaves of *B. darwinii* plants growing at forest edges and below the canopy than in leaves of gap plants (*P* = 0.003; Table 2), while stomatal conductance (*g*_s_) was higher (*P* < 0.001) in leaves of gap plants than in the other two light environments (Table 2).

### SLA, leaf mass per area (LMA) and shoot biomass

Specific leaf area differed significantly for plants from the gap (51.33 ± 1.73 cm^2^ g^−1^), the forest edge (77.48 ± 3.33 cm^2^ g^−1^) and growing under the canopy (122.27 ± 10.12 cm^2^ g^−1^), as did LMA (*P* ≤ 0.001; Table 1). Stem biomass of current-year shoots did not differ between plants growing in the three light environments studied (Table 1). The lowest leaf biomass value was found in shoots of plants growing below the canopy (Table 1).

## Discussion

### Growth and biomass allocation of *B. darwinii* shoots under different light conditions

The variation in physiological and morphological traits of *B. darwinii* plants growing under different light conditions at the study sites provides information on their degree of acclimatization to contrasting environments. Plant architecture depends on endogenous growth processes but is affected by the environment (Barthélémy and Caraglio 2007). At sites with similar soil moisture content, light availability differences influence *B. darwinii* branching abundance. Since plants growing in sunny environments produce more shoots than those in intermediate and low light, leaf exposure is reduced. Shoot morphology also shows differences, since *B. darwinii* plants growing in the gap evade full sunlight by producing shorter shoots with smaller internode length than in the other environments studied. According to Valladares and Pearcy (1998), great differences can be found in overall plant architecture depending on whether they should maximize capture or avoid excess of light. Light capture also depends on leaf display angles, leaf anatomy and other morphological and physiological traits (Givnish 1988; Gutschick 1999) not considered in the present work. Although *B. darwinii* plants growing under the canopy are less branched, longer shoots allow spatial exploration, producing the same leaf number but with lower biomass investment. Low leaf biomass and LMA could be achieved by low mesophyll density or the presence of thin cell walls, although Lusk *et al.* (2008) found different patterns in seedlings. However, Poorter (2007) indicates that these variables are dependent on variations in plant ontogeny from seedlings to adult plants. With regard to the increase in SLA values from gaps to under the canopy, *B. darwiini* plants follow the typical intra-specific variation due to declining light availability (Poorter 1999; Valladares *et al.* 2000).

The higher variability in shoot production, growth and morphology of *B. darwinii* plants present at the forest edge could be caused by the lower homogeneity of light availability in this transitional environment.

Comparing the morphological and functional traits of several invading species, Lamarque *et al.* (2011) found that growth rate was more linked to tree species invasiveness than other traits, such as seedling survival, density, biomass and seed germination. The information on the growth of *B. darwinii* adult plants in their native area will be a valuable contribution to future studies in invasive areas.

### Leaf photosynthesis—light response

In the gap environment *B. darwinii* leaves lost more water but *P*_max_ and *P*_mass_ were higher than in shade plants. Consequently, lower water-use efficiency does not affect assimilation rates in gap plants. In contrast, in species with other life forms in the southern temperate forests in Chile, Saldaña *et al.* (2007) found higher WUE_i_ in plants growing in gaps than under the canopy.

Wright *et al.* (2004) showed that leaf mass per area (LMA) is strongly correlated with photosynthetic capacity on mass bases. Differences in both LMA and SLA between *B. darwinii* plants growing in gaps and at the forest edge are not in concordance with similar and high leaf photosynthesis on mass bases. This result shows that leaves of gap and forest edge plants have similar resources allocated to symplastic components, but gap plant leaves would have a higher proportion of structural components. Both components contribute to leaf construction, allowing adaptation to different light environments (Lusk and Warton 2007). Plants living under the canopy have higher SLA but lower photosynthesis (*P*_max_ and *P*_mass_), approximately half the value of gap and forest edge plants, but they tolerate low light. Nevertheless, as observed at different sites and during successive years, only *B. darwinii* plants growing below the canopy produce no flowers (M.S., unpubl. res.), so that the fixed carbon is dedicated to vegetative growth alone.

Our results allow us to establish that in its native range *B. darwinii* is a species with the ability to grow under different light conditions by adjusting leaf morphology and physiological attributes, but its under canopy performance is lower.

### Comparison of traits between native and invasive ranges

Contrary to our hypothesis, which proposed that the values for the studied variables would differ in *B. darwinii* populations of native and invasive areas, leaf photosynthetic activity determined in the present work shows similar values to those reported by McAlpine *et al.* (2008) in the New Zealand invasion area under similar conditions of canopy openness. These authors determined *P*_max_ values of around 18 µmol CO_2_ m^−2^ s^−1^ in plants growing in full sun and 5 µmol CO_2_ m^−2^ s^−1^ in shade plants, while *g*_s_ was 0.22 mol H_2_O m^−2^ s^−1^ and 0.05 mol H_2_O m^−2^ s^−1^ respectively for sun and shade plants. Furthermore, both in New Zealand and in the native area studied, *B. darwinii* plants showed lower WUE_i_ under full light as a result of higher stomatal conductance. It is important to note that in the invasion area the maximum photosynthetic rate of *B. darwinii* is almost double that of native species in sun, but is similar to all the other species in shade, and stomatal conductance is higher than coexisting native species in the latter environment (McAlpine 2005; McAlpine *et al.* 2008). Inter-species differences could be the main factor affecting exotic species' success.

The only difference observed when the information obtained in native areas is compared with invaded areas is that in the latter the SLA of shade plants (200 cm^2^ g^−1^; McAlpine *et al.* 2008) is higher than in plants of the native area. This would explain why, according to Allen (1991) and Allen and Wilson (1992), *B. darwinii* is one of the few naturalized shrubs in New Zealand that can become established and persist under the canopy. This fact supports the idea that an increase in SLA promotes the invasiveness of exotic plants (Lake and Leishman 2004).

Plants of *B. darwinii* growing in different light environments show similar physiology in their native and invasive ranges. This means that for *B. darwinii*, intra-specific variation of the functional traits studied is not an indicator of successful invasion in new areas.

## Sources of Funding

This work was supported by Universidad Nacional del Comahue. M.S. received a doctoral fellowship from Consejo Nacional de Investigaciones Científicas y Tecnológicas (CONICET).

## Contributions by the Authors

M.S. was involved in planning and performing the experiments, the data analyses and manuscript writing. M.A.D. contributed to manuscript writing. K.D.L. and S.A.V. participated in performing the experiments and data analysis. D.B. was involved in relevant research discussions with other authors.

## Conflicts of Interest Statement

None declared.
